# QuickStats

**Published:** 2013-02-01

**Authors:** Lindsey I. Jones, Jeannine S. Schiller

**Figure:**
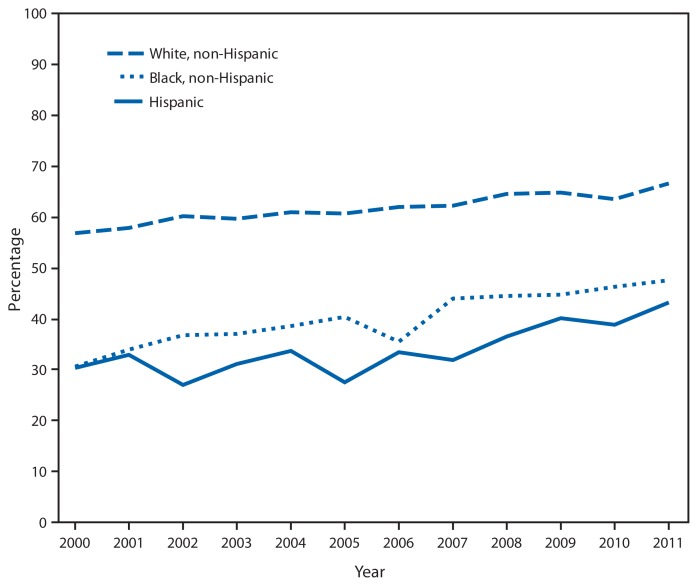
Percentage of Adults Aged ≥65 Years Who Had Ever Received a Pneumococcal Vaccination,^*^ by Selected Race/Ethnicity^†^ — National Health Interview Survey, United States, 2000–2011^§^ ^*^Based on a survey question that asked respondents, “Have you ever had a pneumonia shot? This shot is usually given only once or twice in a person’s lifetime and is different from the flu shot. It is also called the pneumococcal vaccine.” Unknowns were not included in the denominators when calculating percentages. ^†^Persons of Hispanic ethnicity might be of any race or combination of races. ^§^Estimates were based on household interviews of a sample of the U.S. civilian, noninstitutionalized population included in the National Health Interview Survey.

The percentage of adults aged ≥65 years who had ever received a pneumococcal vaccination increased from 56.8% in 2000 to 66.5% in 2011 among non-Hispanic whites, from 30.5% in 2000 to 47.6% in 2011 among non-Hispanic blacks, and from 30.4% in 2000 to 43.1% in 2011 among Hispanics. Throughout 2000–2011, the percentage who had ever received a pneumococcal vaccination was higher among non-Hispanic white adults aged ≥65 years than among Hispanics and non-Hispanic blacks.

**Source:** National Health Interview Survey, 2001–2011 sample adult core component. Available at http://www.cdc.gov/nchs/nhis.htm.

